# Does reflection have an effect upon case-solving abilities of undergraduate medical students?

**DOI:** 10.1186/1472-6920-12-75

**Published:** 2012-08-13

**Authors:** Sebastiaan Koole, Tim Dornan, Leen Aper, Albert Scherpbier, Martin Valcke, Janke Cohen-Schotanus, Anselme Derese

**Affiliations:** 1 Centre for Educational Development, Faculty of Medicine and Health Sciences, Ghent University, Ghent, Belgium; 2 Department of Educational Development and Research, Faculty of Health, Medicine and Life Sciences, Maastricht University, Maastricht, The Netherlands; 3 Institute for Medical Education, Faculty of Health, Medicine and Life Sciences, Maastricht University, Maastricht, The Netherlands; 4 Department of Educational Studies, Faculty of Psychology and Educational Sciences, Ghent University, Ghent, Belgium; 5 University of Groningen and University Medical Centre Groningen, Centre for Research and Innovation in Medical Education, Groningen, The Netherlands

## Abstract

**Background:**

Reflection on professional experience is increasingly accepted as a critical attribute for health care practice; however, evidence that it has a positive impact on performance remains scarce. This study investigated whether, after allowing for the effects of knowledge and consultation skills, reflection had an independent effect on students’ ability to solve problem cases.

**Methods:**

Data was collected from 362 undergraduate medical students at Ghent University solving video cases and reflected on the experience of doing so. For knowledge and consultation skills results on a progress test and a course teaching consultation skills were used respectively. Stepwise multiple linear regression analysis was used to test the relationship between the quality of case-solving (dependent variable) and reflection skills, knowledge, and consultation skills (dependent variables).

**Results:**

Only students with data on all variables available (n = 270) were included for analysis. The model was significant (Anova F(3,269) = 11.00, p < 0.001, adjusted R square 0.10) with all variables significantly contributing.

**Conclusion:**

Medical students’ reflection had a small but significant effect on case-solving, which supports reflection as an attribute for performance. These findings suggest that it would be worthwhile testing the effect of reflection skills training on clinical competence.

## Background

Reflection is a metacognitive process triggered by experience and characterized by three sub-processes: Awareness of self and the situation; critical analysis and understanding of self and the situation; development of new perspectives to inform future actions [1-4]. Reflection on professional experiences is considered to be an attribute that allows healthcare practitioners to cope with demanding and complex professional situations [5-8]. Accordingly, the ability to reflect is identified in many guidelines as an important learning outcome for physicians in training [9-11]. It is proposed that reflection gives a comprehensive view of contextual factors that affect clinical decisions, helps practitioners identify gaps in personal knowledge, and gives direction to their personal development [1,5,12,13]. Unreflective practitioners have been reported to perpetuate routine behaviours and not open them to discussion, have narrow perspectives on their practice, find it difficult to identify learning goals and accept feedback, and find it difficult to adapt their practice [5,13,14]. Accordingly, systematic reflection is seen as essential for continuing professional development and lifelong learning [7,14]. Despite this recognition, however, there is a lack of empirical evidence proving it is indeed effective [2,15].

In the past decade, evidence has been published showing a link between personal attributes and the ability to reflect. Mamede and Schmidt [16] found a negative correlation between reflective practice and a physician’s age and working experience, which they attributed to older and more experienced physicians being more likely to find situations routine and use automatic reasoning based on recognition and instant retrieval of similar situations. Boenink et al. [17] assessed reflection by means of written answers to vignettes. Undergraduate medical students who were female, had previous health care work experience, and who were aiming for careers in general practice tended to have higher reflection scores. The authors concluded that the ability to reflect is trait-like but affected by learning processes. Results based on a self-report questionnaire developed by Sobral et al. [18] showed a relation between reflection and the perceived meaningfulness of learning, which is a marker of the depth of learning. Qualitative studies by Sargeant et al. [19,20] showed that reflection helps learners to accept feedback and use it in their future clinical practice. We found only one study that demonstrated a direct link between reflection and performance. Sobral et al. [21] reported undergraduate students’ scores on a reflection-in-learning scale were significantly, but weakly, correlated with grade point averages, which they used as an indicator of academic achievement.

**Figure 1:**
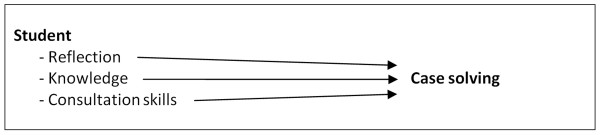
Conceptual research framework.

Given the paucity of evidence linking reflection to student performance, we set out to investigate the effect of reflection on the ability to solve clinical problems. Previous studies found clinical problem solving to be determined by generalizable competence in consultation skills, such as history taking, communication and physical examination and content related competence directed by knowledge [22,23]. To acknowledge these factors and investigate their interaction with reflection we included the latter two as independent variables in a study, which set out to answer the question: What effect does reflection add to the knowledge and consultation skills on students’ case solving? (Figure1).

## Methods

### Participants

At Ghent University, undergraduate medical students follow a seven year integrated contextual curriculum, comprising patient centred, student centred, community orientated, problem based and evidence based education [24]. The first two and a half years focus on the healthy and normal body and continue in a second cycle of two and a half years to address the body systems from a clinical perspective. Year six comprises rotational clerkships and year seven is a transitional year to postgraduate education. In the present study data was collected among students in the second cycle during year 2008–2009 (n = 362).

### Method/instruments

In line with the conceptual research framework, data were collected on the four variables shown in Figure1- the quality of case solving, reflection skills, level of knowledge and level of mastery of consultation skills. The variables “case-solving” and “reflection” were measured by presenting each student with two interactive video-cases, which confronted participants with authentic clinical problems in a standardized assessment context [25]. They showed a simulated patient consulting a physician with a new clinical problem. Scenes were filmed from the physician’s perspective, to make the cases as realistic as possible. All consultations had the same structure: 1) reason for encounter; 2) history; 3) physical examination; 4) explanation of diagnosis, advice and treatment planning; and 5) closing the consultation. Each consultation was interrupted six times with a question (e.g. “What would you ask?”,”What physical examinations would you suggest?”, “Explain the diagnosis to the patient?”) against an otherwise blank screen. To mirror real-life consultations where there is limited time to think, a countdown timer showed students how long they had left to respond.

#### Quality of case solving

Students’ case-solving was measured by comparing their answers with a list of expected ones. Case scripts and evaluation forms were authored by the same two skills lab teachers to ensure coherent scoring. To test interrater reliability, three assessors (the skills lab teachers and SK) independently scored 30 student reactions per case. A Krippendorff’s alpha coefficient [26] above 0.97 for each case showed that interrater reliability was high so the remaining answers were single-rated. Respondents’ total score over the two cases was then the variable ‘quality of case solving’.

#### Reflection skills

Immediately after solving the video-cases, students were asked to reflect on the video-cases and their reactions to the six questions they had been asked. Because the structure of reflective comments varies so much between individuals [1,27], six questions were used to structure their responses into six reflection skills related to the three main elements of reflection: awareness; understanding; and future action (Table 1). Reflection skills were scored using the 6-item Student Assessment of Reflection Scoring rubric (StARS®), which has been demonstrated as a valid instrument for reflection in undergraduate medical students at Ghent University [28]. StARS® provides assessors with quality definitions for all items [29,30], which are scored on 0–5 scales. All items together form an overall reflection score. All reflections were assessed by SK, who computed the variable ‘reflection skills‘- the aggregate of overall reflection scores on both cases.

**Table 1:** The referred reflection skills related to the three key elements in the six questions to structure student reflections

***Key element***	***Reflection skills***
*Awareness of the experience*	*1: The ability to describe an experience adequately. 2: The ability to identify essential elements and describe own thoughts and feelings.*
*Understanding the experience*	*3: The ability to pose searching questions.4: The ability to answer searching questions and being aware of the relevant frames of reference.*
*Impact on future actions*	*5: The ability to draw conclusions.6: The ability to describe concrete learning goals and plans for future action.*

#### Level of knowledge

Knowledge was measured by performance in the Dutch inter-university progress test, which assesses knowledge across all medical disciplines at the level of exit from the undergraduate curriculum [31], during the same academic year as case solving and reflection were assessed. The progress test is a valid and reliable indicator of knowledge acquisition for undergraduate medical students in the Netherlands [32]. It has also been validated in the context of the undergraduate medical curriculum at Ghent University [24].

#### Mastery level of consultation skills

Consultation skills are taught in a continuing strand - clinical, technical and communication skills – that runs through years 4–6 of our medical programme. Consultation skills, communication skills, and technical skills are examined using multiple tests: Clinical skills are assessed with and without simulated patients by four experienced physicians in a four station objective structured clinical examination; communication skills are assessed by two communication experts in a specific consultation setting with simulated patients; and technical skills are assessed by a written test of rational prescribing and a computer test of ordering and interpreting medical imaging. Scores from those examinations are combined into a single score, representing the generic skills needed to perform a consultation. To identify the mastery level of consultation skills at the same point in a student’s trajectory as the other variables included in this study, the single course scores of the years 2008–2009 were used for analysis.

### Analysis

Stepwise multiple linear regression analysis was used to determine the predictive value of reflection scores, knowledge, and consultation skills on video-case solving, which was the dependent variable. The stepwise regression procedure aimed to produce a parsimonious model, explaining the dependent variable by including or excluding predictor variables stepwise. The contribution of each variable to the model, its significance level, and the variance explained by the whole model are reported. All statistical analyses were performed using SPSS 17.0 (SPSS Inc, Chicago, IL, USA) with a pre-set significance level of p ≤ 0.05.

## Results

Two hundred and seventy students (75 % of the total student population) had data on case solving, reflection, knowledge, and consultation skill scores and were therefore eligible to be included in the analysis. Missing data were caused by timetable clashes, illness, and other factors which were unlikely to have a systematic effect on the findings. Table 2 shows descriptive statistics for all variables included in the analysis.

**Table 2:** Descriptive statistics of all variables (highest possible score) in the multiple linear regression analysis; Mean, Standard deviation (SD), Minimum (Min) and Maximum (Max)

**Variables**	**Mean**	**SD**	**Min**	**Max**
*Dependent*				
Case solving score (20)	10.0	2.3	4.3	15.6
*Independent*				
Knowledge score (100)	35.0	8.3	6.3	62.9
Consultation skill score (20)	13.7	2.1	0.0	17.0
Reflection score (60)	38.6	7.5	16.0	54.0

There were only weak correlations (Pearson r < 0.30) between the independent variables, confirming they were distinct constructs. Multiple linear regression analysis resulted in a significant model (Anova F (3,269) = 11.00 and p < 0.001) with an adjusted R square of 0.10. The model and its coefficients are described in Table 3.

**Table 3:** The Beta values (B), Standard Error (SE B) and the Standardized Beta (β) of all coefficients in the linear regression analysis model, based on all students

**Coefficient**	**B**	**SE B**	**β**
Constant	3.94	1.10	
Knowledge score	0.04	0.02	0.16^*^
Consultation skill score	0.17	0.07	0.15^*^
Reflection score	0.06	0.02	0.19^**^

Note: ^*^ p ≤ 0.05, ^**^ p ≤ 0.01.

## Discussion

Medical students’ ability to reflect was a significant, albeit weak, predictor of the quality of their case solving after allowing for the effects of knowledge and consultation skills. That is in line with findings of Sobral [21] demonstrating a significant but weak correlation (r = 0.21, p = 0.003) between undergraduate medical students’ scores on a reflection-in-learning scale and academic achievement. He explained this relationship by the underlying metacognitive skills of reflection, which also affect academic achievement through learning. A similar explanation can also be applied to the present study. Reflection includes the ability to relive an experience, analyze it critically, and come up with conclusions after careful exploration of alternatives [13,16,33]. Using such skills might have helped students with high reflection scores to understand the case content more profoundly and to give more carefully considered answers, which resulted in higher case solving scores.

Our results demonstrate that case solving both triggers and is affected by reflection. This relationship, however, is not as circular as it might appear. At its heart lies a distinction between the content and process of reflection. Whereas the content of reflection is context specific and influenced by its triggering experience and learners’ unique frame of reference, the process of reflection has a more generic character [34,35]. In the present study, case solving as a triggering experience is related to the content of reflection. The effect of reflection on case-solving that we found, however, refers to the process of reflection, which is driven by more generic reflective skills.

Focus on those generic skills makes it possible to assess reflections while recognizing the uniqueness of both a learner’s frame of reference and the context in which their reflection was initiated [4]. It also provides a counter-argument to the argument that our results can be accounted for by having measured reflection skills and the quality of case-solving in the same context whilst knowledge and consultation skills were assessed in a different context. The focus on process skills made the influence of context less important.

Although the predictive effect of reflection, knowledge and consultation skills on the quality of case solving was statistically significant, the model only explained 10 % of the total variance. From previous studies we would have expect the levels of knowledge and consultation skills to account for more variance than was demonstrated here [22,23]. First, this inconsistency with earlier studies may be explained by the different methods used to assess case solving. As opposed to answering questions in video-cases, other studies used objective structured clinical examinations (OSCE) derived formats as clinical performance examinations (CPX) and Integrated Structured Clinical Exams (ISCE). These methods required practical knowledge and executive skills and are called performance assessment in vitro whereas video-based approach in the present study exampled a clinical context based test where students had to demonstrate theoretical knowledge by means of writing skills [36]. Second, the specific indicators of knowledge and consultation skills may have contributed to the modest explained total variance of our model. The Dutch inter-university progress test is designed to test a greater breadth of knowledge than was needed to solve the questions in the video-cases [24]. The scores students received in the course ‘clinical, technical and communicative skills’, used as variable for consultation skills, also included competence in radiology and pharmacology next to consultation and communication skills. Whilst these broader aspects of competence were not included in previous studies, they were clearly relevant to the diagnostic and treatment planning aspects of the video-cases.

The modest total of variance explained by our regression model suggests the set of three predictors in the model was incomplete. Factors such as case difficulty, the time of testing, and test environment were similar for all students; personal factors, however, could make cases more or less difficult for individual students and contribute to variance in the scores. Desmedt [37] identified motivation, beliefs, and self-efficacy as relevant factors, alongside gender, personality, intelligence and learning style. Future research could address limitations of the current study by developing a more comprehensive model to describe case-solving. It could also test the generalizability of our findings to a workplace context and from case scores to clinical practice.

## Conclusion

Undergraduate medical students’ reflection had a small but significant effect on the quality of case solving. This empirical finding suggests that helping students develop their ability to reflect might be beneficial and it would therefore be worth testing the effect of reflection skills training on clinical competence.

## Competing interests

The authors declare that they have no competing interests.

## Authors’ contributions

SK, AD and MV conceptualized the idea, SK and LA were involved in collecting data and SK, AD and TD were involved in writing the initial drafts. All authors were involved in the revising drafts and made essential contributions to this paper and critically reviewed and approved the final manuscript.

## Pre-publication history

The pre-publication history for this paper can be accessed here:

http://www.biomedcentral.com/1472-6920/12/75/prepub
